# A methodological approach to improve extracellular staphylococcal clearance in internalization assays using bone-associated host-cell models of orthopedic infections

**DOI:** 10.3389/fcimb.2026.1945457

**Published:** 2026-09-28

**Authors:** Stefania Mamberti, Giada Meogrossi, Elena De Vecchi, Vincenzo Pennone, Arianna B. Lovati

**Affiliations:** 1 IRCCS Galeazzi - Sant’Ambrogio Hospital, Cell and Tissue Engineering Laboratory, Milan, Italy; 2 Fondazione Istituto Insubrico Ricerca per la Vita, Gerenzano, Italy; 3 IRCCS Galeazzi - Sant’Ambrogio Hospital, Laboratory of Clinical Chemistry and Microbiology, Milan, Italy

**Keywords:** antimicrobials, bacterial internalization, lysostaphin, multiplicity of infection, orthopedic infections, *Staphylococcus aureus*, *Staphylococcus epidermidis*, vancomycin

## Abstract

Orthopedic implant-related infections are a major cause of prosthesis failure and are associated with biofilm formation, antimicrobial tolerance, and bacterial persistence. Bacterial internalization into bone-associated host cells may also contribute to chronic or recurrent infection. However, non-standardized extracellular bacterial clearance, multiplicity-of-infection definition, and data normalization limit assay interpretation and comparability. This study aimed to establish a stepwise protocol with a structured strategy to obtain the extracellular staphylococcal clearance allowing for the unbiased quantification of host-cell internalized bacteria through identification and prevention of major methodological pitfalls. Cell-associated bacteria from host-cell lysates were a secondary endpoint. Methicillin-resistant *S. aureus* USA300 and *S. epidermidis* RP62A were used as model pathogens, whereas MG-63 osteosarcoma cells and human bone-marrow mesenchymal stromal cells served as host-cell models. Vancomycin and lysostaphin were first evaluated in the absence of host cells with bacterial inocula simulating multiplicities of infection (MOI) of 50 and 100. Clearance was more effective and consistent at the lower inoculum. Accordingly, 25 µg/mL lysostaphin for 30 min and 100 µg/mL vancomycin for 60 min were tested individually or sequentially after a 2-h infection at an MOI of 50. Vancomycin alone resulted in the highest residual extracellular bacterial recovery, precluding interpretation of CFU recovered from cell lysates. Lysostaphin-containing protocols reduced viable CFU recovered from the final treatment medium to near or below the assay detection limit. *S. epidermidis* recovery from cell lysates was negligible after lysostaphin-containing treatments. Phase-contrast microscopy monitored host-cell morphology, whereas confocal microscopy supported intracellular bacterial localization. These findings indicate that antimicrobial treatments should be validated within the bacteria–host-cell system rather than selected solely from cell-free assays. The framework integrates strain-specific antimicrobial testing, inoculum selection, verification of extracellular clearance, normalization to host-cell number, and microscopy-based assessment, improving the rigor and comparability of *in vitro* studies of staphylococcal interactions with bone-associated cells.

## 1 Introduction

Orthopedic implant-related infections remain a major cause of implant failure despite continuing advances in research to improve osseointegration and antimicrobial agents (Li et al., 2025; Seidelman and DeBaun, 2025). Orthopedic infections can originate through intraoperative inoculation or, more rarely, through hematogenous seeding (Smith and Chetter, 2015; Uçkay et al., 2009; Rakow et al., 2019). A central reason for this clinical difficulty is the ability of staphylococci to evade both antibiotics and host defenses through two complementary strategies. One key mechanism is biofilm formation on the prosthesis surface, where bacteria adopt slow-growing or metabolically altered states, promoting infections that are clinically silent, difficult to diagnose and unresponsive to systemic antibiotic treatment (Arciola et al., 2018). These infections may require prolonged antimicrobial treatment, surgical debridement, implant revision or removal, thereby adversely affecting patients’ quality of life and increasing healthcare costs (Kurtz et al., 2014; Tande and Patel, 2014). Beyond biofilm formation, an additional and less understood mechanism may further contribute to the persistence and recurrence of orthopedic infections: the internalization of bacteria into the bone-associated host cells, where they can evade both the immune system (Collet et al., 2025) and conventional antibiotic treatments (Josse et al., 2015). Although intracellular persistence has been proposed as a potential reservoir for recurrent infection, its contribution to orthopedic implant failure remains incompletely defined. Addressing this question first requires experimental methods that reliably distinguish internalized bacteria from residual extracellular or extracellularly-adherent bacteria.

Among staphylococcal species implicated in orthopedic infections, *S. aureus* is the best-known for invading non-phagocytic host cells, including osteoblasts, where it can persist intracellularly and drive chronic, relapsing infections (Josse et al., 2015; Mouton et al., 2021). In contrast, *S. epidermidis*, the coagulase-negative species most frequently isolated in device-related infections (Severn and Horswill, 2023), invades host cells far less efficiently, via still poorly defined mechanisms (Campoccia et al., 2016). To our knowledge, no broadly accepted protocol has been established across staphylococcal strains and non-professional phagocytic host-cell models, and published assays vary substantially in MOI definition, extracellular-clearance procedures, and CFU normalization (Marro et al., 2021; Stracquadanio et al., 2021). A crucial parameter of any internalization experiment is the multiplicity of infection (MOI), the ratio of bacteria added to host cells. Reported MOIs range from 0.5:1 to 500:1 depending on the bacterial species and study, often calculated on the seeded cell number at the time of plating rather than on the actual cell count at the time of infection (Tricou et al., 2024; Mouton et al., 2021), which can differ substantially when infection is performed 24–48 hours after seeding. The reporting method chosen for expressing the colony-forming unit (CFU) counts is another parameter which crucially affects results interpretation and the possibility to compare different bacterial species and experimental studies. Currently, the literature inconsistently reports CFU counts in the different forms of absolute values and normalized counts per fixed or infected host-cell numbers, without always stating which reporting method was used (Jevon et al., 1999; Hamza and Li, 2014; Bongiorno et al., 2020). Reliable interpretation requires direct microbiological verification of residual viable extracellular bacteria and an independent imaging approach to assess bacterial localization relative to host-cell boundaries. Critically, many studies do not explicitly verify that extracellular bacteria have been fully eliminated by the antimicrobial treatment before quantifying internalized bacteria and simply rely on literature-based evidence of antimicrobial efficacy (Tricou et al., 2024; Mouton et al., 2021). Only a few research groups verify extracellular clearance during the internalization experiment and still confirm intracellular localization by an independent method such as microscopy (Hamza and Li, 2014). Antibiotic-protection assays rely on the assumption that the selected treatment eliminates extracellular and cellular surface-adherent bacteria without affecting host-cell integrity or the viability and recovery of intracellular bacteria. Violation of either assumption can substantially bias the apparent internalization rate. The gentamicin protection assay is widely used to eliminate extracellular bacteria because gentamicin generally shows limited short-term penetration into mammalian cells. Nevertheless, its intracellular uptake and activity may vary depending on concentration, exposure duration, and host-cell type. Under some experimental conditions, including infected osteoblast models, gentamicin can reduce the recovery of internalized bacteria and potentially underestimate the intracellular population (Kim et al., 2019; Rigaill et al., 2021; Yu et al., 2020). Therefore, gentamicin-based assays should be validated within each specific bacteria–host-cell system. These inconsistencies hinder the comparability and reproducibility of internalization data across studies and represent the central rationale behind the present work. The primary method-validation question of this study was whether an antimicrobial treatment could reduce extracellular and cellular surface-adherent *S. aureus* USA300 and *S. epidermidis* RP62A to a level that would permit reliable interpretation of viable bacteria recovered from host-cell lysates. Since *S. epidermidis* RP62A is known to be gentamicin resistant, the antibiogram of the two strains was assessed during this study. The primary endpoint was residual viable extracellular bacterial recovery after antimicrobial treatment. Secondary endpoints were cell-associated CFU recovered from host-cell lysates, CFU normalized to host-cell number, and microscopy-based assessment of bacterial localization relative to host-cell boundaries. We systematically investigated the conditions for internalization experiments of staphylococcal pathogens relevant to orthopedic infections. The selected conditions were applied to the osteosarcoma cell line MG-63 and to primary human bone marrow mesenchymal stromal cells (hBM-MSCs) infected with each strain. This study aimed to establish a stepwise protocol to identify robust and model-specific experimental conditions for bacterial internalization studies, with a focus on orthopedic infections but potentially applicable to other research fields involving host-pathogen interactions.

## 2 Materials and methods

### 2.1 Materials and equipment

#### 2.1.1 Bacterial strains and culture reagents

Methicillin-resistant *Staphylococcus aureus* TCH1516 [USA300-HOU-MR] (ATCC BAA-1717) and methicillin-resistant, biofilm-forming *Staphylococcus epidermidis* RP62A (ATCC 35984) were obtained from the American Type Culture Collection (ATCC, Manassas, VA, USA). The strains are hereafter referred to as *S. aureus* and *S. epidermidis*, respectively. The following reagents were used for bacterial culture: Tryptone (tryptic) Soy Agar (TSA), #10195, Lickson Srl, Pettineo, Italy; Brain Heart Infusion (BHI), #1.10493.0500, Millipore^®^, and vancomycin hydrochloride, #PHR1732, Supelco^®^, both purchased from Merck Life Science, Milan, Italy; lysostaphin, #HY-P2329, MedChemExpress MCE, D.B.A. Italia Srl, Segrate, Italy; 0.9% NaCl saline, #B100406, Fresenius Kabi Italia Srl, Isola della Scala, Italy.

#### 2.1.2 Mammalian cells and culture reagents

Bone-associated cell models: primary human Bone-Marrow-derived Mesenchymal Stromal Cells (hBM-MSCs) (Poietics™ hMSC; #PT2501, Lonza Group Ltd, Basel, Switzerland; lot no. 24TL007134, 24TL249163, 25TL064141); human osteosarcoma MG-63 cell line (ATCC^®^ CRL-1427™). The following reagents were purchased from Gibco, Life Technologies Italia, Milan, Italy: Minimum Essential Medium Eagle – Alpha Modification (alpha-MEM), #22571-020; Dulbecco’s Modified Eagle Medium High Glucose (DMEM HG), #10938-025; 4-(2-HydroxyEthyl)-1-PiperazineEthaneSulfonic acid (HEPES), #15630-056; Penicillin-Streptomycin (PS), #15140122; Penicillin-Streptomycin-Glutamine (PSG), #10378-016; Phosphate Buffered Saline (PBS), #10010023; 0.5% trypsin-Ethylenediaminetetraacetic acid (EDTA), #15400-054. Additional reagents: Fetal Bovine Serum (FBS), #SV3016003, Cytiva HyClone; Fibroblast Growth Factor 2 (FGF-2), #100-18B, Peprotech. Cell culture medium for hBM-MSC: alpha-MEM, supplemented with 10% FBS, 10 mM HEPES, 1% PS, 5 ng/mL FGF-2. Cell culture medium for MG-63 cells: DMEM HG supplemented with 10% FBS, 1% PSG.

#### 2.1.3 Immunostaining reagents

Antibodies used for *S. epidermidis* detection: mouse IgG3 anti-*Staphylococcus epidermidis* primary monoclonal antibody (clone 17-5), #MA1-35788, Invitrogen; goat anti-mouse IgG3 secondary polyclonal antibody, Alexa Fluor^®^ 488, #A-21151, Invitrogen, Thermo Fisher Scientific, Waltham, MA, USA. Antibodies used for *S. aureus* detection: rabbit IgG anti-*S. aureus* primary polyclonal antibody, #ab20920, Abcam; goat anti-rabbit IgG H&L secondary polyclonal antibody Alexa Fluor^®^ 488, #ab150077, Abcam, Cambridge, UK. Additional reagents: Poly-D-Lysine hydrobromide (PDL), MW 70000-150000, #HY-139201A, MedChemExpress MCE^®^, D.B.A. Italia S.R.L., Segrate, Italy; Paraformaldehyde (PFA) solution 4% in PBS, #sc-281692, ChemCruz, Santa Cruz Biotechnology, Dallas, USA; Bovine Serum Albumin (BSA), #A7906, and Triton™ X-100, #T8787, Sigma-Aldrich^®^, Merck Life Science, Milan, Italy; Alexa Fluor^®^ 647-conjugated Wheat Germ Agglutinin (WGA-AF647), #W32466, Invitrogen, Thermo Fisher Scientific, Waltham, MA, USA; Tween^®^ 20, #A4974, PanReac AppliChem, ITW Reagents S.R.L., Monza, Italy; DAPI, #ab228549, and anti-fade fluorescence mounting medium, #ab104135, Abcam, Cambridge, UK.

#### 2.1.4 Equipment

Implen OD600^®^ photometer (Implen, Munich, Germany). Vitek2 Compact system (Biomérieux, Craponne, France). Phase-contrast Olympus CKX53-SLP microscope (Olympus, Tokyo, Japan) with a 40× air objective, equipped with the Olympus EP50 camera (Olympus, Evident Technology Center Europe, Münster, Germany). Leica TCS SP8 confocal microscope equipped with an HC PL APO CS2 (63× magnification/1.40 numerical aperture) oil-immersion objective and a Leica DFC9000 GTC camera (Leica Microsystems, Wetzlar, Germany); the following lasers with the corresponding target excitation (ex)/emission (em) wavelengths were used in sequential scanning mode: Diode 405 laser (ex 385/em 460) for DAPI detection; OPSL 488 laser (ex 485/em 545) for the detection of the AF488-conjugated secondary antibodies against *S. aureus* and *S. epidermidis*; Diode 638 laser (ex 630/em 680) for AF647-WGA detection; Leica Life Science Application Suite X (LAS X) software (Leica Microsystems, Wetzlar, Germany) for image acquisition and 3D volume reconstructions. Fiji software (version 1.54p, NIH, USA http://imagej.org, Java 21.0.7 64-bit) for image processing. GraphPad Prism software version 8.0.2 for Windows (GraphPad Software, Boston, MA, USA).

### 2.2 Methods

#### 2.2.1 Bacterial culture

*S. aureus* and *S. epidermidis* were stored at −20 °C in bacterial growth medium supplemented with 20% glycerol and were freshly grown by streaking an aliquot from the stocks onto TSA plates with an inoculating loop; plates were then incubated overnight at 37°C and subsequently stored at +4 °C until use, up to one week. The day before experiments, a single colony was picked up with an inoculation loop from the above-described plates, inoculated in 10 mL BHI broth and incubated overnight. Subsequent dilution of the overnight culture was also performed in BHI broth. All bacterial cultures were performed at 37 °C.

#### 2.2.2 Mammalian cell culture

Bone-associated cell models, hBM-MSCs and MG-63 cells, were routinely subcultured at 70-80% confluence by splitting through trypsinization, subsequent counting at a Bürker counting chamber and resuspension in supplemented medium. Cells from three donors were pooled in equal proportions to generate a standardized composite cell preparation. Donor-specific variability could therefore not be estimated. Cells were grown at 37 °C in a humidified atmosphere containing 5% CO_2_.

#### 2.2.3 Bacterial antibiogram, growth kinetics and OD_600_-to-CFU calibration

The phenotypic resistance to antibiotics of *S. aureus* and *S. epidermidis* was determined with the automated system Vitek2, using the antimicrobial susceptibility testing (AST) card for Staphylococci and/or Enterococci, following the manufacturer’s instructions.

To standardize the inoculum used in all subsequent infection assays, two complementary parameters were defined for *S. aureus* and *S. epidermidis*: the culture time required to reach mid-log phase, and the respective OD_600_ absorbance corresponding to a defined cell density. Both were determined starting from an overnight culture preparation. A single colony of each strain was inoculated into 10 mL BHI broth and incubated overnight at 37 °C; overnight cultures were then pelleted (4000 × g, 15 min, RT) and resuspended in sterile saline.

For growth kinetics, the saline suspension was adjusted to 0.5 McFarland turbidity (corresponding nominally to ~1.5 × 10^8^ CFU/mL; the actual concentration was subsequently determined by CFU enumeration) and diluted 1:100 in fresh BHI broth. At defined time points (0, 2, 3, 4, 5, and 6 h), serial tenfold dilutions (10^–1^ to 10^–6^) were prepared in saline and 10 µL of each dilution was plated in quintuplicate on TSA. After overnight incubation at 37 °C, Colony-Forming Units (CFU) were enumerated. Growth curves were generated from the mean CFU ± SD of two independent experiments (each with n = 5 technical replicates per time point) for each strain. Under the specified culture conditions, the 3-h time point (T3) fell within the exponential-growth interval and was selected as the operational infection time point.

OD_600_ absorbance of the selected time points was correlated with CFU/mL to enable rapid preparation of inocula at a higher resolution. OD_600_ measurements were performed using saline as the blank. The saline suspension of the overnight pellet was adjusted to different OD_600_ absorbances (*S. aureus*: 0.25, 0.35, 0.45; *S. epidermidis*: 0.35, 0.45), then diluted 1:100 in 10 mL of fresh BHI and grown for 3 h to reach the mid-log phase T3 time point, as established through the growth curves. T3 cultures were then centrifuged (4000 × g, 15 min, RT), resuspended in saline, and again adjusted across a range of OD_600_ values (*S. aureus*: 0.30, 0.35, 0.40, 0.45, 0.50, 0.55; *S. epidermidis*: 0.45, 0.50, 0.55, 0.60) to identify the inoculum density. At each OD_600_ tested, serial tenfold dilutions were prepared in saline, and 10 µL of each dilution was plated in triplicate on TSA for CFU enumeration. After overnight incubation at 37 °C, colonies were counted, and CFU/mL was calculated using the dilution factors. On this basis, the OD_600_ values selected to obtain infection-ready mid-log (T3) cultures were 0.25 (*S. aureus*) and 0.45 (*S. epidermidis*) upon resuspension of the overnight culture, and 0.55 (*S. aureus*) and 0.60 (*S. epidermidis*) upon resuspension of the T3 culture, corresponding to 1.33 × 10^8^ and 2.5 × 10^8^ CFU/mL, respectively.

#### 2.2.4 Optimization of a cell-free bacterial clearance assay

This initial screening was performed to evaluate the ability of lysostaphin and vancomycin to reduce recoverable bacteria under two defined bacterial-burden conditions. The purpose of this experiment was to identify candidate antimicrobial concentrations and exposure times for subsequent validation in the complete bacteria–host-cell system.

Bacterial suspensions were prepared as described above to obtain T3 mid-log-phase cultures. The inocula were prepared at densities equivalent to MOIs of 100:1 or 50:1 (from here onwards referred to as inocula equivalent to nominal MOIs of 100 and 50) based on a simulated infection of 5 × 10^4^ cells per well. An aliquot of the adjusted OD_600_ saline suspension was therefore transferred to 10% FBS DMEM (without antibiotics) at a bacterial concentration corresponding to 10 × 10^6^ (MOI 100) or 5 × 10^6^ (MOI 50) CFU/mL, before distributing 500 μL per well (5 or 2.5 × 10^6^ CFU/well, respectively) in 24-well plates. After 2 hours of incubation at 37°C, the wells were washed twice with 500μL PBS to assess the clearance of plastic surface-adherent bacteria. The remaining bacteria were then exposed to either lysostaphin at final concentrations of 10, 25, or 50μg/mL or vancomycin at final concentrations of 50 or 100μg/mL, prepared by diluting stock solutions in DMEM supplemented with 10% FBS, and incubated for 30min or 1hour at 37°C. Three wells per experiment were used as untreated controls. After treatment, whole culture volumes (500 µL) were collected, wells were washed twice with 500 µL PBS, and residual adherent bacteria were resuspended in 100 µL saline. Both the whole culture volumes and the residual bacteria collected in saline were serially diluted 10^–1^ to 10^-6^ in saline and plated on TSA for CFU determination after overnight incubation at 37 °C. Total surviving bacteria per well were calculated as the sum of the bacteria recovered from the treatment medium and from the adherent-bacterial fraction (for a total of 500 µL + 100 µL = 600 µL). The limit of detection (L.o.D.) of bacterial count was set at 100 CFU/mL and adjusted to the final 600 µL collection volume to a L.o.D. = 60 CFU. Percent survival was then calculated by normalizing the total surviving bacteria to the untreated control. Each condition was tested in n=6 and n=9 independent experiments for MOI 100 and MOI 50, respectively.

#### 2.2.5 Infection of eukaryotic mammalian cells

For infection experiments, MG-63 cells and hBM-MSCs were seeded in 24-well plates at densities of 4 × 10^4^ and 4.5 × 10^4^ per well, respectively, in the corresponding complete growth medium. After 24 h, cells from three wells were detached and counted with a hemocytometer to determine the mean of host cell number at the time of infection. Bacterial infections were performed separately for each strain following the same experimental procedure. For each independent experiment, a fresh overnight bacterial culture was used to prepare a 3-h mid-logarithmic-phase culture as described above. The OD_600_-adjusted bacterial suspensions were diluted in antibiotic-free DMEM or α-MEM supplemented with 10% FBS to obtain a nominal MOI of 50, corresponding to approximately 2.5 × 10^6^ bacteria per 5 × 10^4^ host cells in a final volume of 500 µL per well. The actual bacterial inoculum used in each independent experiment was verified by serial dilution and CFU enumeration. The actual MOI was calculated by dividing the measured bacterial inoculum by the mean host-cell number determined at the time of infection. Infection was performed for 2 h at 37 °C in a humidified atmosphere containing 5% CO_2_. Three types of control were run in parallel for each cell type and bacterial strain in the corresponding antibiotic-free medium (DMEM for MG-63, α-MEM for hBM-MSCs): (i) a host cells control, consisting of uninfected host cells cultured without exposure to bacteria and collected before the 2 h-infection; (ii) a bacterial control, consisting of the bacterial inoculum incubated without host cells and collected after 2 h; and (iii) a bacteria-host cell unit, consisting of host cells that were exposed to bacteria for 2 hours without receiving any subsequent antimicrobial treatment and collected after the 2 h-infection. None of the controls were exposed to antimicrobials. All controls were harvested and processed in the same way as the treated samples. After the 2-h incubations, the whole culture volume of 500 µL was collected from every control well, serially diluted in saline and plated on TSA; subsequently, wells were washed twice with PBS, and controls containing host cells were subjected to osmotic lysis in 100 µL cold sterile Milli-Q water for 20 min, while bacteria-only control wells were rinsed in 100 µL saline. In both cases, the 100 µL volumes were serially diluted in saline and plated on TSA. All TSA plates were grown overnight at 37 °C for CFU enumeration. After the 2-h infection, infected cells were washed twice with PBS and incubated with antimicrobials to eliminate extracellular bacteria. Four extracellular-clearance treatments were tested to identify the optimal protocol for eliminating bacteria: lysostaphin alone (25 µg/mL, 30 min), vancomycin alone (100 µg/mL, 1 h), and the two sequential combinations (lysostaphin followed by vancomycin; vancomycin followed by lysostaphin). Sequential treatments were separated by two washes with 500 µL PBS. Plates were examined by phase-contrast microscopy before and after antimicrobial exposure to document host-cell morphology, confluence, treatment-associated cell detachment and the infection process. At the end of the complete treatment, the final treatment medium was collected for the enumeration of residual extracellular bacteria. Collected samples were serially diluted in sterile saline and plated on TSA using the drop-plate method. Cells were then washed twice with PBS and incubated in 100µL of cold sterile Milli-Q water for 20min to induce osmotic lysis. Lysates were resuspended, serially diluted with saline (10^–1^ to 10^–6^), and plated on TSA. After overnight incubation at 37 °C, colonies were enumerated. Based on a set limit of detection (L.o.D.) of bacterial count of 100 CFU/mL, the L.o.D. was adjusted to the respective collection volume (i.e. L.o.D. = 100 CFU/mL x collection volume; 50 CFU for bacteria derived from the whole culture volume of 500 µL; 10 CFU for bacteria derived from the 100 µL lysate volume and expressed as CFU per well; 0.2 CFU for bacteria derived from the 100 µL lysate volume and expressed as CFU/10^3^ cells). CFUs recovered from host-cell lysates were reported as cell-associated CFUs and normalized to the mean host-cell number determined at the time of infection. These values were interpreted as intracellular only when extracellular clearance and intracellular localization were independently demonstrated. Each condition was assessed in 12 independent experiments.

#### 2.2.6 Immunostaining

Confocal Laser Scanning Microscopy (CLSM) was used to qualitatively assess the localization of bacteria relative to host-cell boundaries. For this purpose, n = 3 independent experiments were performed in which cells were seeded on Ø12 mm glass coverslips 24 h before the infection assay. To improve hBM-MSC adherence, the corresponding coverslips were previously coated with poly-D-lysine (0.01% PDL in ddH_2_O) through incubation overnight at 37 °C, 5% CO_2_, subsequent removal, double wash with ddH_2_O and air drying. This coating was not necessary for the MG-63 cell line. After performing the internalization experiment as described above, at the end of each treatment, cells were washed twice with PBS, fixed for 15 min with 4% paraformaldehyde (PFA) at room temperature, and washed again twice with PBS. The cellular membrane was permeabilized for 15 min with 0.5% Triton X-100 to allow the entry of antibodies. After three 5-min washes in PBS, blocking was performed by incubating samples with 1% Bovine Serum Albumin (BSA) in PBS for 30 min at room temperature. Subsequently, a 1-h incubation with antibodies was performed in a dark, humidified staining chamber at room temperature.

For the *S. aureus* internalization experiment, samples were first incubated with the primary rabbit IgG anti-*S. aureus* polyclonal antibody; after one 5-min wash in PBS and two 10-min washes in 0.01% Tween 20 to remove nonspecific binding, samples were incubated with the Alexa Fluor^®^ 488-conjugated secondary goat anti-rabbit IgG H&L polyclonal antibody.

For the *S. epidermidis* internalization experiment, samples were first incubated with the primary mouse IgG3 anti-*Staphylococcus epidermidis* monoclonal antibody; after one 5-min wash in PBS and two 10-min washes in 0.01% Tween 20 to remove nonspecific binding, samples were incubated with the Alexa Fluor^®^ 488-conjugated secondary goat anti-mouse IgG3 polyclonal antibody. All antibodies were diluted in 1% BSA to a final concentration of 2.5 µg/mL. In both cases, secondary antibody incubation was followed by the aforementioned washing protocol. Cellular membranes were then stained with 5 µg/mL of Alexa Fluor^®^ 647-conjugated Wheat Germ Agglutinin (WGA-AF647, diluted from stock in PBS) for 20 min at room temperature. After an additional 25-min wash, nuclei were counterstained with 5 µg/mL DAPI for 10 min at room temperature. The excess salts were removed by dipping cells in ddH_2_O. Cells were mounted with 10 µL of aqueous anti-fade mounting medium on glass slides and sealed with nail polish. Samples were stored at -20 °C until imaging. Stained samples were imaged using a confocal laser microscope in sequential scanning mode. An optical volume of approximately 7-15 µm was imaged with a 0.3 µm z-step size to encompass the whole host-cell volume (pinhole: 1.00 Airy Units; line average: 2). The LAS X software was used to produce reconstructions of the 3D volumes. Acquisitions were exported as LIF files and processed with the Fiji software for the creation of image panels.

#### 2.2.7 Statistical analysis

The independent experiment, run using independently prepared bacterial and host cell cultures, was the experimental unit. CFU values were analyzed on the log_10_scale. Data below the assay limit of detection were assigned a value equivalent to the L.o.D. prior to logarithmic transformation and statistical analysis. Data from the cell-free assay for antimicrobial clearance were shown to be normally distributed using the Shapiro-Wilk test. Statistical significance was assessed using the Two-Way ANOVA with the assumption of sphericity, followed by Tukey’s multiple-comparisons test. For the cell-based internalization assays, statistical analysis was performed using the Kruskal–Wallis test followed by Dunn’s test for multiple comparisons, as the data were not normally distributed. Data are presented as individual independent-experiment values as the mean ± SEM, together with the individual experimental values. P values < 0.05 were considered statistically significant. Statistical analyses were performed using the GraphPad Prism software.

## 3 Results

### 3.1 Bacterial antibiotic resistance, growth kinetics and OD_600_-to-CFU calibration

The antibiograms showed that *S. epidermidis* was resistant to penicillin, gentamicin, erythromycin, linezolid and trimethoprim/sulfamethoxazole, while *S. aureus* was resistant to penicillin and erythromycin. Since *S. epidermidis* was confirmed resistant to gentamicin, for experimental consistency between the two bacterial species, vancomycin, one of the most used antibiotics in orthopedic infections involving methicillin-resistant staphylococci, was used in this work.

To identify the mid-log phase, the growth curve of both *S. aureus* and *S. epidermidis* was analyzed. As shown in **Figure 1**, CFU/mL increased progressively over time for both S. *aureus* and S. *epidermidis*, from approximately 5 x 10^5^ CFU/mL at time 0, followed by a steady increase at each subsequent time point, reaching approximately 10^9^ CFU/mL by T6. The two strains displayed a similar growth trend and the time point of 3 hours (T3) was identified as mid-log phase.

**Figure 1:**
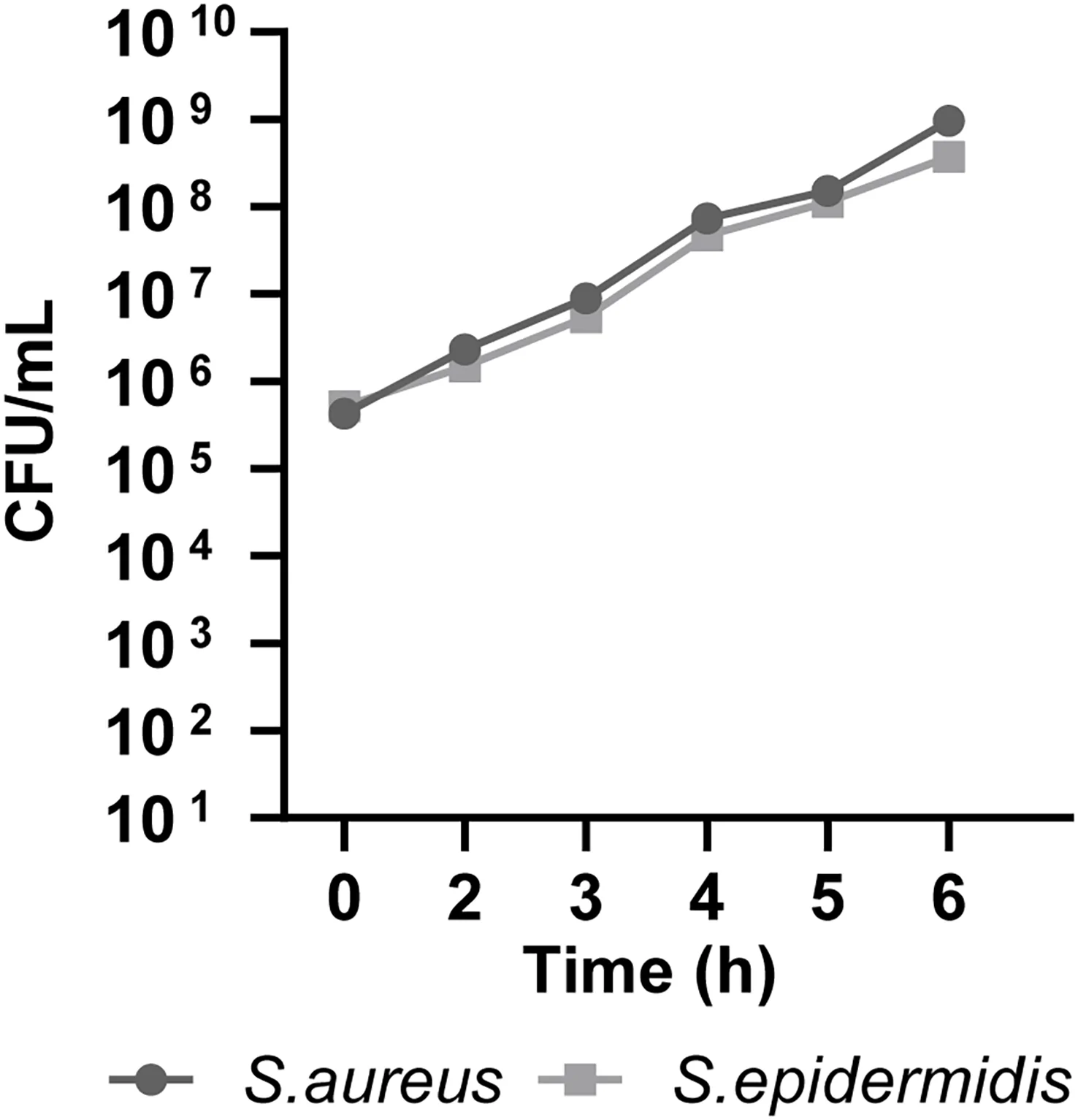
Growth curves of *S. aureus* and *S. epidermidis*. Data are shown as the result of two independent experiments for each strain.

Subsequently, the bacterial density was corrected through OD_600_ measurements after both overnight culture and after T3 culture, to optimize the OD_600_-to-CFU calibration for the subsequent infection experiments. The following OD_600_ values were identified for an optimized 1:100 dilution of the overnight culture: 0.25 for *S. aureus*; 0.45 for *S. epidermidis*, respectively corresponding to a mean of 1.31 x 10^8^ CFU/mL and 3.77 x 10^8^ CFU/mL. At mid-log-phase, the following OD_600_ values were identified for an optimized inoculum of infection: 0.55 for *S. aureus*; 0.60 for *S. epidermidis*, respectively corresponding to 1.33 × 10^8^ CFU/mL and 2.5 × 10^8^ CFU/mL.

### 3.2 Optimization of the cell-free bacterial clearance assay

After defining mid-log phase and OD_600_–CFU calibration, two antimicrobials were tested on both strains in a cell-free model to identify optimal concentrations and exposure times. Susceptibility at different simulated MOIs assessed extracellular clearance in subsequent infection experiments.

#### 3.2.1 Antimicrobial treatment of bacteria at a simulated MOI of 100

For each strain, bacterial populations at a simulated MOI of 100 were treated with either 50 or 100 µg/mL vancomycin for 30 or 60 min (**Figure 2A**). Vancomycin treatment significantly reduced bacterial counts compared with the untreated control for both *S. aureus* and *S. epidermidis* (****, p < 0.0001; n = 6). For *S. aureus*, bacterial loads remained between 5.4 and 5.8 log_10_ CFU after exposure to 50 or 100 µg/mL vancomycin for 30 or 60 min, compared with approximately 6.9 log_10_ CFU in the control. The corresponding survival percentages were approximately 9.5% and 7.2% after 30 min, and 7.6% and 4.0% after 60 min, at 50 and 100 µg/mL, respectively. The greatest reduction was observed at 100 µg/mL after 60 min, although the overall effect of increasing concentration or exposure time was modest. Similarly, *S. epidermidis* counts were reduced to approximately 5.5–5.8 log_10_ CFU, with no clear concentration-dependent or time-dependent improvement. The corresponding survival percentages were approximately 9.8% and 8.3% after 30 min, and 8.1% and 21.2% after 60 min, at 50 and 100 µg/mL, respectively.

**Figure 2:**
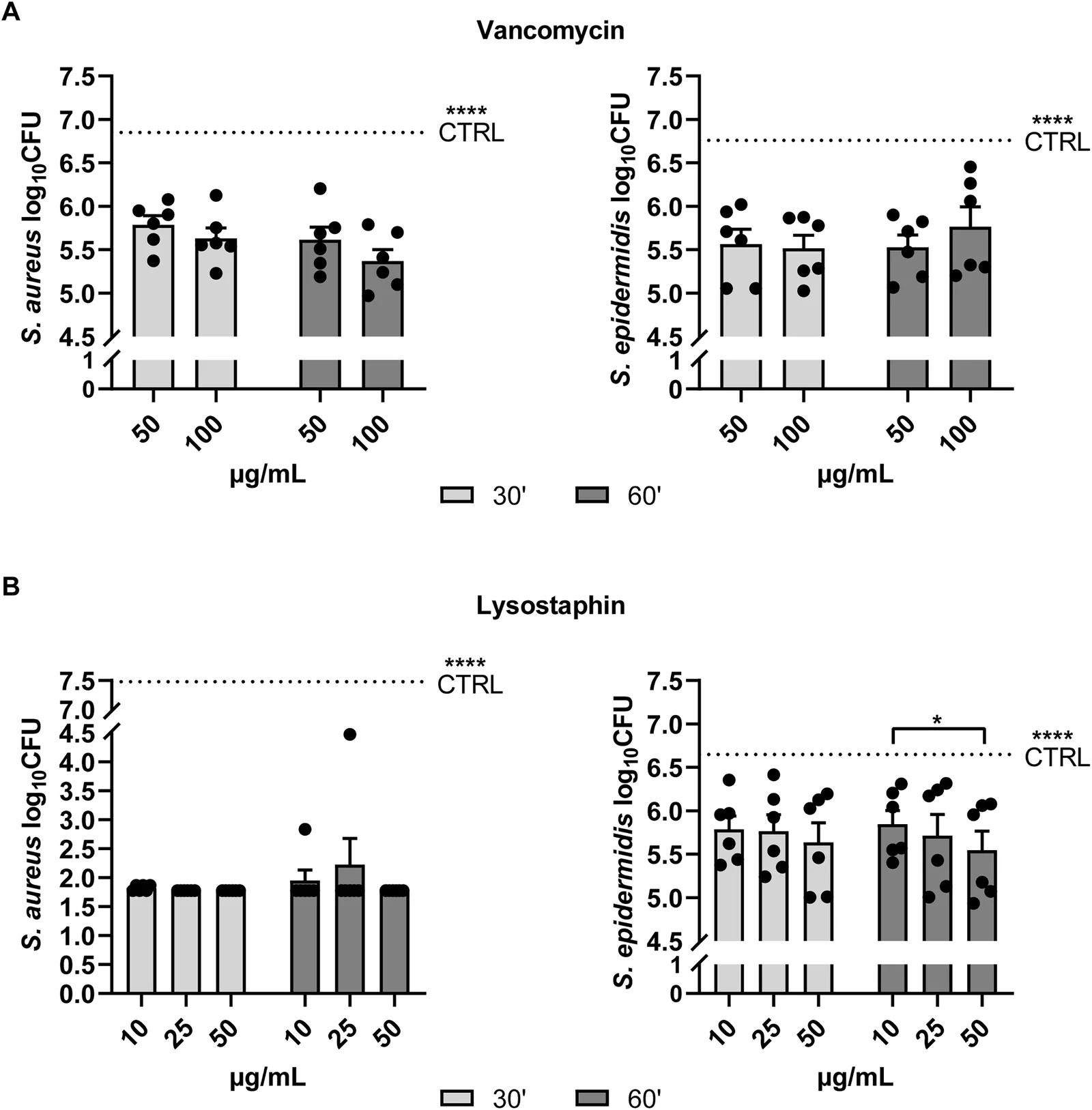
Antimicrobial clearance of bacteria in the cell-free assay at a simulated MOI of 100. *S. aureus* (left) and *S. epidermidis* (right) under simulated MOI of 100 conditions after **(A)** treatment with either 50 or 100 µg/mL vancomycin for 30 or 60 minutes or **(B)** 10, 25, or 50 µg/mL lysostaphin for 30 or 60 minutes. L.o.D.: 60 CFU. Data are shown as mean ± SEM (n = 6); ****p<0.0001; *p<0.05.

Similarly, lysostaphin was tested at 10, 25 and 50 µg/mL (**Figure 2B**). Lysostaphin showed markedly greater species-specific activity. In *S. aureus*, treatment reduced counts from approximately 7.5 log_10_ CFU in the control to near or below the detection limit, particularly at 25 and 50 µg/mL. Survival was approximately 0% at 10, 25, and 50 µg/mL after 30 and 60 min. Limited residual growth was observed at 10 µg/mL and, in some replicates, at 25 µg/mL after 60 min. Overall, all lysostaphin treatments produced a highly significant reduction relative to the control (p < 0.0001). In contrast, lysostaphin had a more limited effect on *S. epidermidis*, with mean counts remaining between approximately 5.6 and 5.9 log_10_ CFU. The corresponding survival percentages were approximately 23.4%, 26.4%, and 21.5% after 30 min, and 26.7%, 28.3%, and 17.5% after 60 min, at 10, 25, and 50 µg/mL, respectively. Nevertheless, all treatment conditions were lower than the control (p < 0.0001), and after 60 min, 50 µg/mL resulted in a modest but significant reduction compared with 10 µg/mL (p < 0.05).

#### 3.2.2 Antimicrobial treatment of bacteria at a simulated MOI of 50

Vancomycin treatment of the *S. aureus* population at a simulated MOI of 50 reduced bacterial survival under all tested conditions, although the response was not consistently concentration-dependent (**Figure 3A**). Vancomycin reduced the viable burden of both *S. aureus* and *S. epidermidis*. In *S. aureus*, treatment with 50 and 100 µg/mL resulted in approximately 5.5 and 5.6 log_10_ CFU after 30 min, corresponding to 35.7% and 93.1% survival, respectively, compared with 6.3 log_10_ CFU in the untreated control. After 60 min, counts decreased to approximately 5.1 log_10_ CFU at both concentrations, corresponding to 7.3% and 9.5% survival. This time-dependent reduction was significant at 50 µg/mL (p < 0.01) and 100 µg/mL (p < 0.001), whereas no concentration-dependent effect was observed. In *S. epidermidis*, 50 and 100 µg/mL of vancomycin produced approximately 5.3 and 5.5 log_10_ CFU after 30 min, corresponding to 7% and 12% survival. After 60 min, counts decreased to 4.89 and 4.86 log_10_ CFU, corresponding to 2.3% and 2.0% survival, compared with 6.6 log_10_ CFU in the control. All treatments reduced bacterial counts relative to the control (p < 0.0001), and the effect of increasing exposure time was significant at both 50 µg/mL (p < 0.01) and 100 µg/mL (p < 0.001).

**Figure 3:**
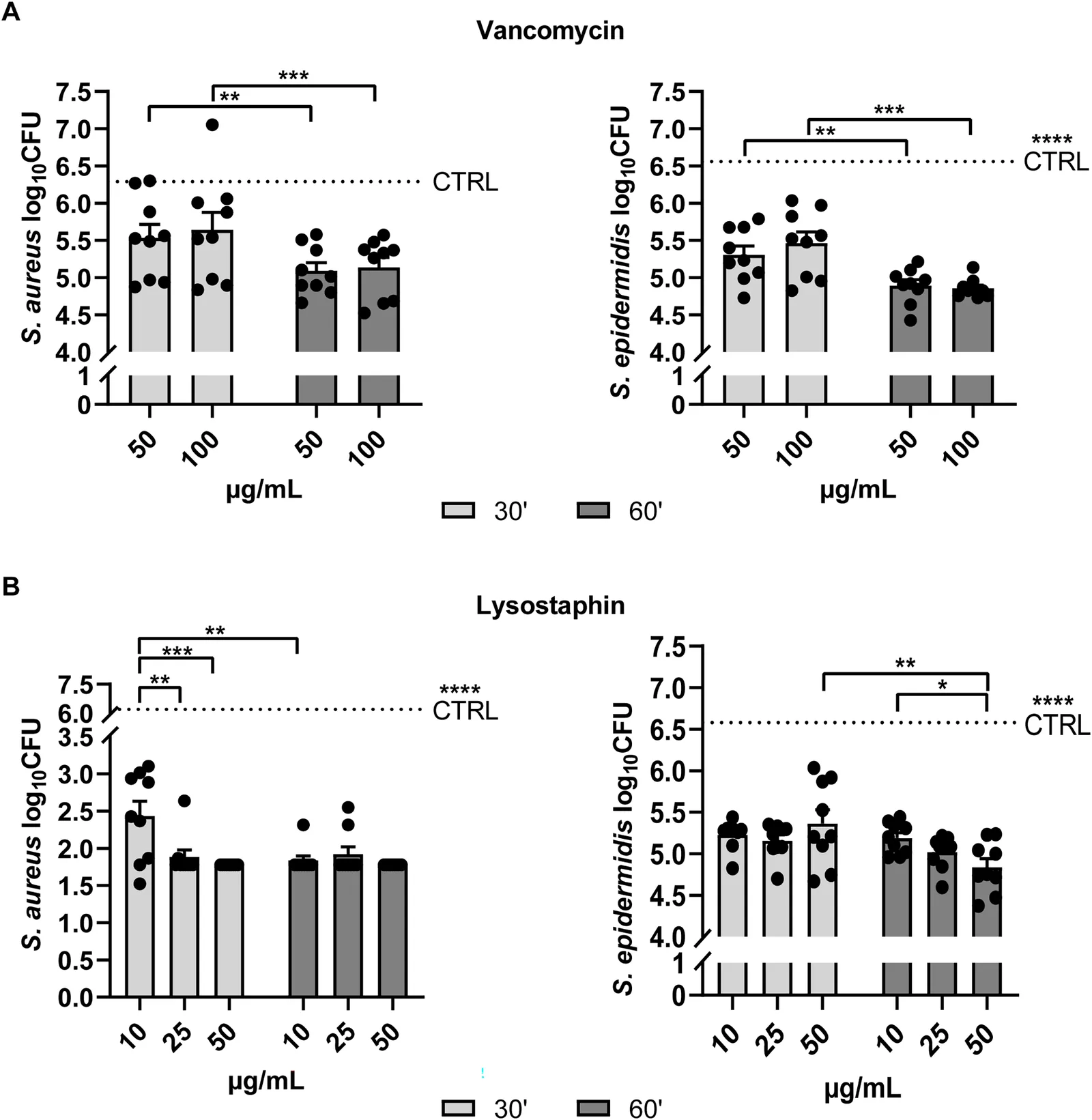
Antimicrobial clearance of bacteria in the cell-free assay at a simulated MOI of 50. *S. aureus* (left) and *S. epidermidis* (right) under simulated MOI of 50 conditions after **(A)** treatment with either 50 or 100 µg/mL vancomycin for 30 or 60 minutes or **(B)** 10, 25, 50 µg/mL lysostaphin for 30 or 60 minutes. L.o.D.: 60 CFU. Data are shown as mean ± SEM (n = 9); ****p<0.0001; ***p<0.001; **p<0.01; *p<0.05.

Lysostaphin showed substantially greater activity against *S. aureus* (**Figure 3B**). After 30 min, 10, 25, and 50 µg/mL produced approximately 2.4, 1.9, and 1.8 log_10_ CFU, corresponding to approximately 0% survival. Counts at 25 and 50 µg/mL were significantly lower than at 10 µg/mL (p < 0.01 and p < 0.001, respectively). After 60 min, survival remained approximately 0% at all concentrations. Extending treatment with 10 µg/mL significantly increased bacterial killing (p < 0.01), and all conditions reduced counts relative to the untreated control measuring 6.2 log_10_ CFU (p < 0.0001). Against *S. epidermidis*, lysostaphin had a more moderate effect. After 30 min, counts ranged from 5.2 to 5.4 log_10_ CFU, corresponding to 4.5%, 3.9%, and 9.8% survival at 10, 25, and 50 µg/mL, respectively. After 60 min, counts were 5.2, 5.0, and 4.8 log_10_ CFU, corresponding to 4.2%, 2.8%, and 2.1% survival, compared with 6.6 log_10_ CFU in the control (p < 0.0001). At 50 µg/mL, the 60-min treatment produced lower counts than the 30-min treatment (p < 0.01), and after 60 min, counts were lower at 50 than at 10 µg/mL (p < 0.05). Overall, prolonged vancomycin exposure enhanced bacterial killing in both species, whereas lysostaphin was markedly more active against *S. aureus* than against *S. epidermidis*.

Based on the overall response profiles, an MOI of 50 was selected for subsequent experiments. This condition provided a sufficiently robust bacterial challenge while preserving a broader and more informative experimental dynamic range. In comparison, the higher bacterial burden at MOI 100 appeared to partially limit treatment efficacy, particularly against *S. epidermidis*. Lysostaphin-treated *S. epidermidis* cultures showed substantially higher residual survival at MOI 100 than at MOI 50, suggesting that the higher inoculum may exceed or approach the effective antibacterial capacity under the tested conditions. Therefore, MOI 50 represents a more suitable compromise between maintaining a biologically relevant bacterial challenge and avoiding an excessive inoculum effect that could mask treatment-dependent differences.

### 3.3 Translation to cell-based internalization assays

The antimicrobial conditions selected in the cell-free assay were validated in bacteria–host-cell co-cultures after a 2-h infection, testing each agent alone and in both sequential combinations to compare their effects on the reduction of bacteria recovered from the final treatment medium and bacterial recovery from the cell-associated fraction.

#### 3.3.1 Recovery of cell-associated *S. aureus* following antibiotic treatment

In *S. aureus* assays, extracellular bacterial recovery varied across treatments, but in hBM-MSCs, no comparison reached statistical significance. Vancomycin alone left the highest residual extracellular load, whereas all lysostaphin-based treatments reduced it to near the L.o.D. (**Figure 4A**). The cell-associated fraction showed the same trend, both as total CFU, with a mean of (4.46 ± 1.24) × 10^6^ CFU (**Figure 4B**), and as CFU normalized per 10³ cells, with a mean of (8.93 ± 2.48) × 10^4^ CFU/10³ cells (**Figure 4C**): vancomycin alone gave the numerically highest recovery and the two sequential combinations (L+V and V+L) the lowest, without significant differences among treatments. In MG-63 cells, these treatment-dependent differences were significant. Vancomycin alone again produced the highest extracellular bacterial recovery (**Figure 5A**), significantly exceeding all other treatments, indicating incomplete reduction of bacteria recovered from the final treatment medium and limiting interpretation of CFU recovered from the cell-associated fraction (**Figures 5B, C**). Lysostaphin alone and both sequential combinations reduced extracellular recovery to near the detection limit and yielded significantly lower cell-associated CFU than vancomycin, with the V+L sequence giving the lowest values (**Figures 5B, C**).

**Figure 4:**
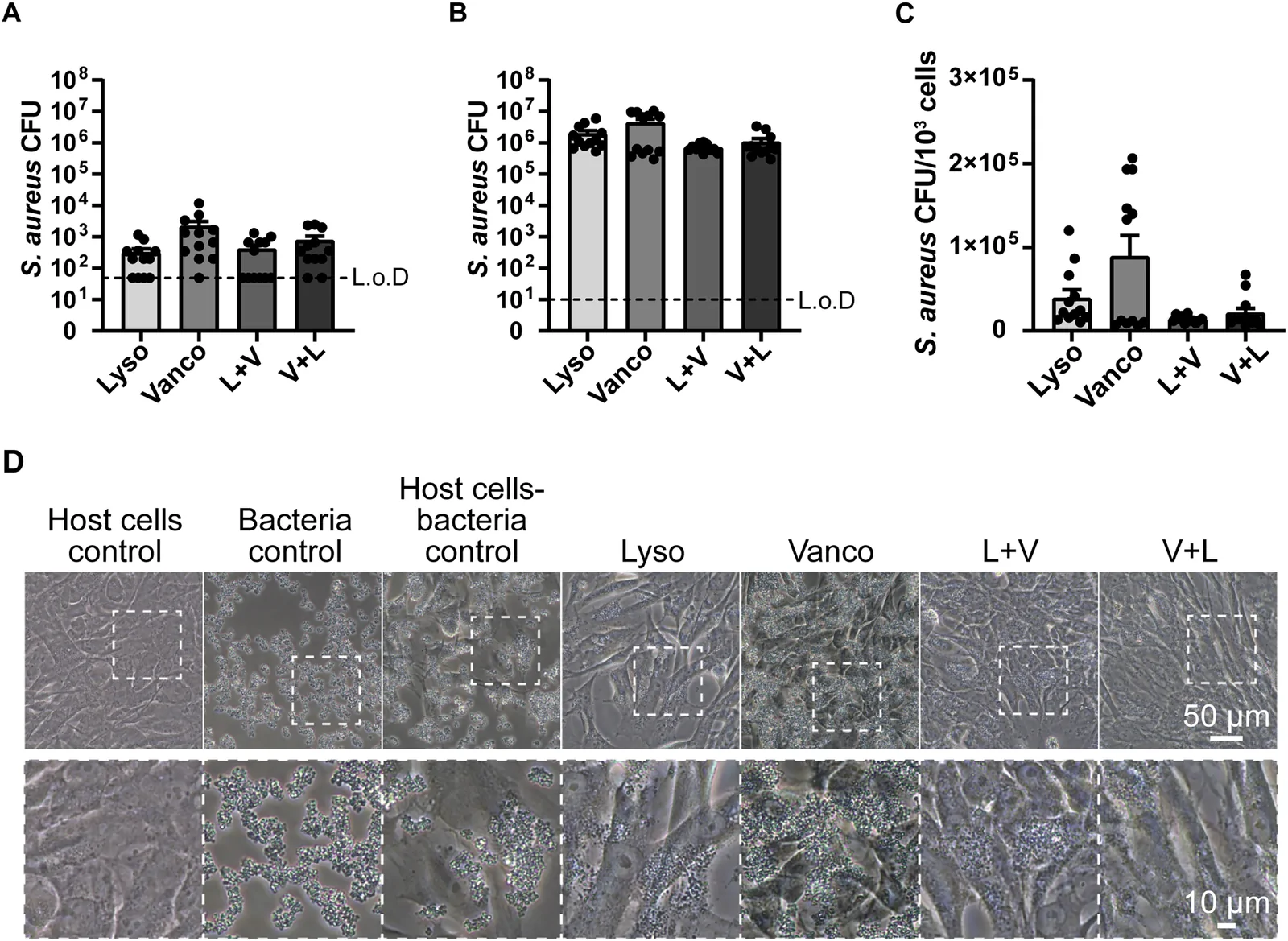
Effect of lysostaphin, vancomycin, and their combination on residual extracellular and cell-associated bacterial recovery of *S. aureus* in hBM-MSCs. **(A)** Total extracellular CFU. L.o.D.: 50 CFU. **(B)** Total cell-associated CFU from cell lysates. L.o.D.: 10 CFU. **(C)** Cell-associated CFU normalized per host cell (CFU/10^3^ cells). L.o.D.: 0.2 CFU/10^3^ cells. **(D)** Representative phase-contrast microscopy images of live cells at each treatment step; bottom panels correspond to enlargements of the dashed white squares in the top panel. Lyso, lysostaphin; Vanco, vancomycin; L+V, lysostaphin followed by vancomycin; V+L, vancomycin followed by lysostaphin. Data are shown as mean ± SEM (n = 12).

**Figure 5:**
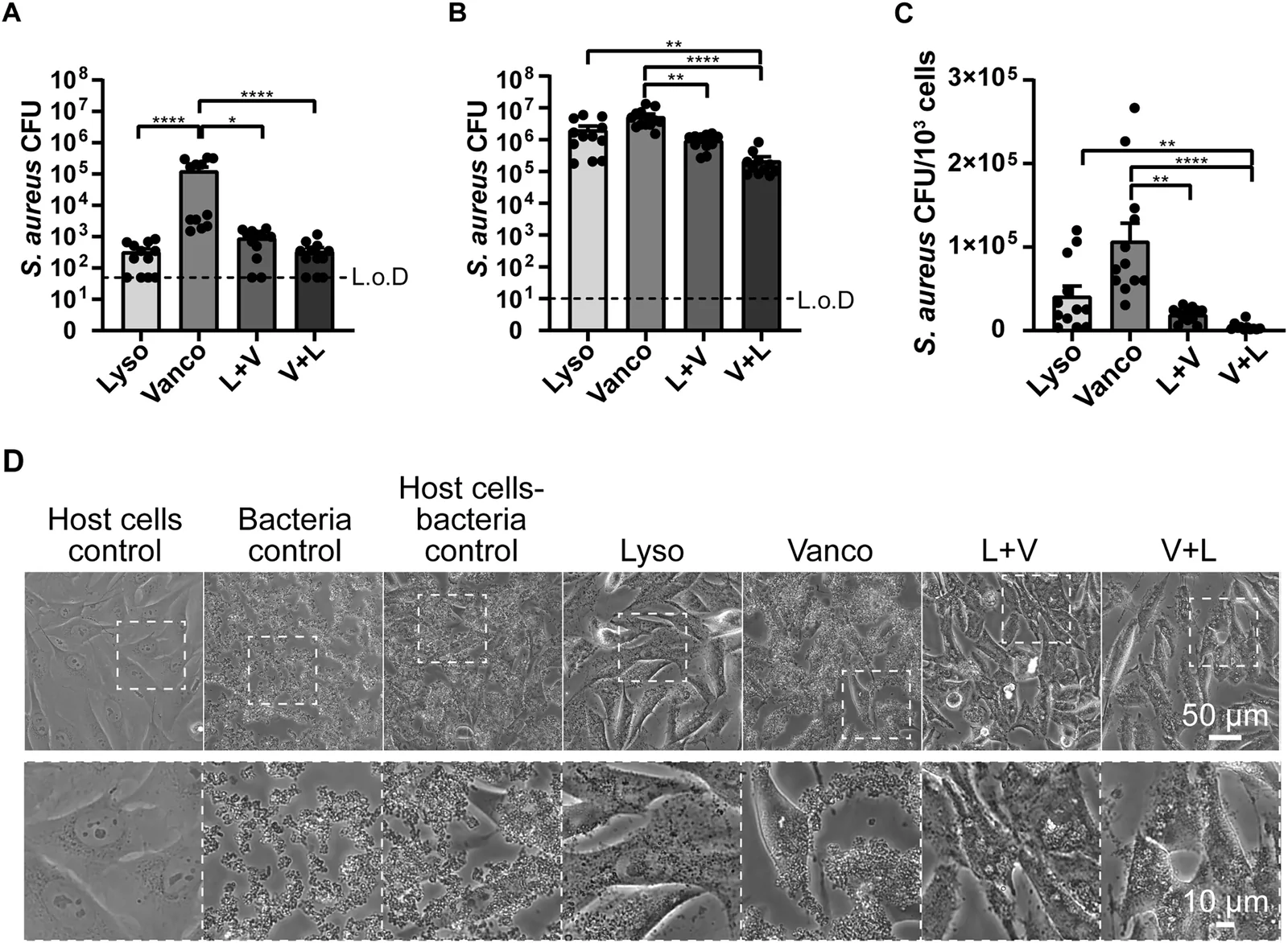
Effect of lysostaphin, vancomycin, and their combination on residual extracellular and cell-associated bacterial recovery of *S. aureus* in MG-63 cells. **(A)** Total extracellular CFU. L.o.D.: 50 CFU. **(B)** Total cell-associated CFU from cell lysates. L.o.D.: 10 CFU. **(C)** Cell-associated CFU normalized per host cell (CFU/10^3^ cells). L.o.D.: 0.2 CFU/10^3^ cells. **(D)** Representative phase-contrast microscopy images of live cells at each treatment step; bottom panels correspond to enlargements of the dashed white squares in the top panel. Lyso, lysostaphin; Vanco, vancomycin; L+V, lysostaphin followed by vancomycin; V+L, vancomycin followed by lysostaphin. Data are shown as mean ± SEM (n=12); ****p<0.0001; **p<0.01; *p<0.05.

#### 3.3.2 Recovery of cell-associated *S. epidermidis* following antimicrobial treatment

The same antimicrobial treatments were tested against *S. epidermidis* in both cell types. As observed for *S. aureus*, extracellular and cell-associated recovery varied with treatment. In hBM-MSCs cultures, vancomycin alone yielded the highest extracellular bacterial recovery, remaining clearly above the L.o.D. (**Figure 6A**), whereas lysostaphin alone reduced extracellular CFU to near-background levels and both sequential combination treatments eliminated detectable extracellular bacteria. The cell-associated fraction followed the same pattern seen in *S. aureus*: total (**Figure 6B**) and normalized CFU (**Figure 6C**) were significantly higher when vancomycin was used without any other treatment. All lysostaphin-based treatments remained close to the L.o.D. in absolute counts (**Figure 6B**) and dropped near zero on the normalized scale (**Figure 6C**), with no significant differences among them. A similar pattern was observed in MG-63 cells. Vancomycin alone resulted in the highest extracellular CFU (**Figure 7A**) and the highest bacterial recovery from cell lysates, as a total (**Figure 7B**) and as CFU normalized per 10³ cells (**Figure 7C**), significantly exceeding all other treatments. Lysostaphin alone reduced the extracellular population to near the L.o.D., whereas both sequential combinations lowered it to undetectable levels (**Figure 7A**). Cell-associated recovery for all three lysostaphin treatments remained at or near the L.o.D. (**Figure 7B**) and close to baseline once normalized (**Figure 7C**), with no significant differences among them.

**Figure 6:**
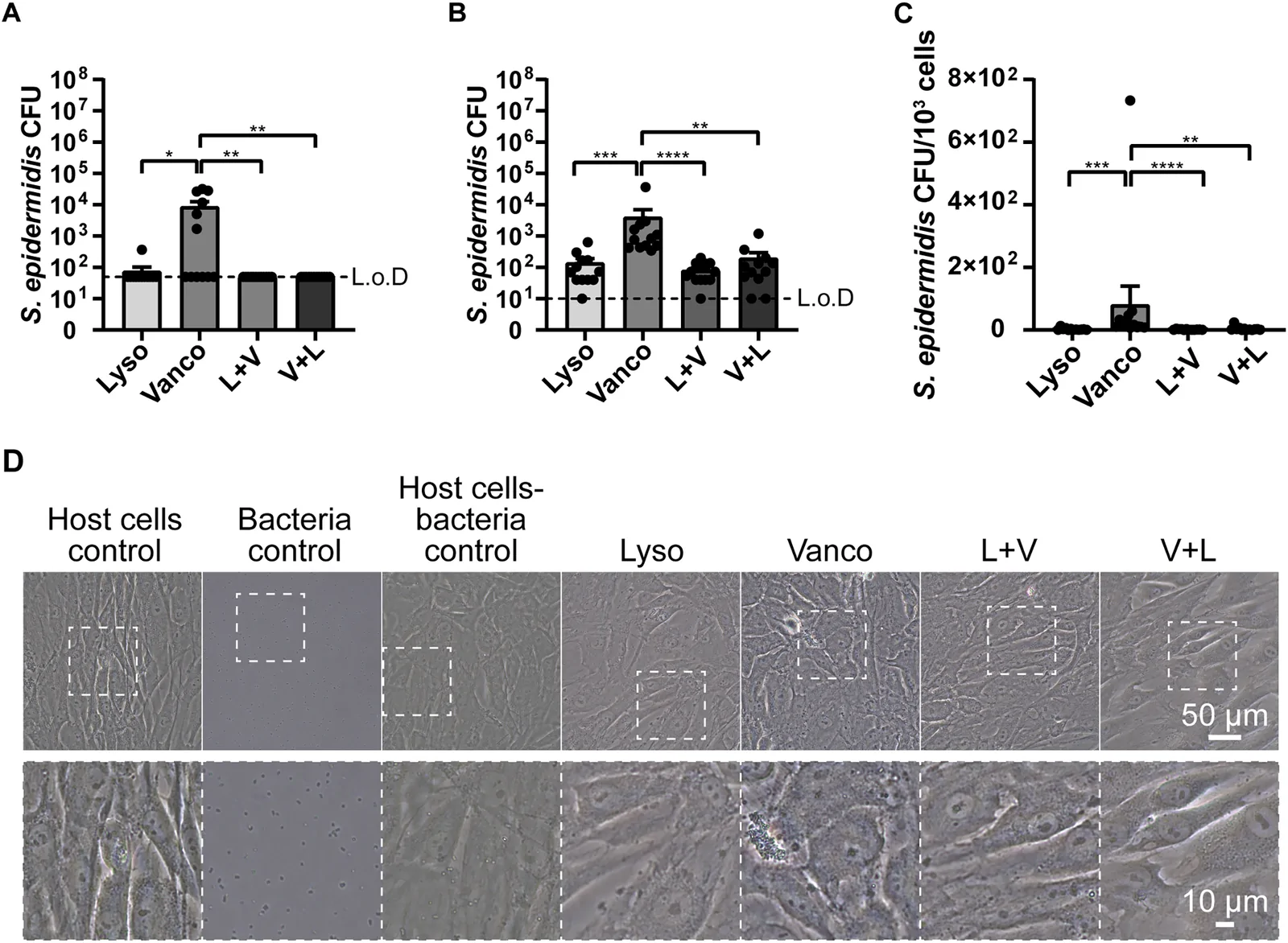
Effect of lysostaphin, vancomycin, and their combination on residual extracellular and cell-associated bacterial recovery of *S. epidermidis* in hBM-MSCs. **(A)** Total extracellular CFU. L.o.D.: 50 CFU. **(B)** Total cell-associated CFU from cell lysates. L.o.D.: 10 CFU. **(C)** Cell-associated CFU normalized per host cell (CFU/10^3^ cells). L.o.D.: 0.2 CFU/10^3^ cells. **(D)** Representative phase-contrast microscopy images of live cells at each treatment step; bottom panels correspond to enlargements of the dashed white squares in the top panel. Lyso, lysostaphin; Vanco, vancomycin; L+V, lysostaphin followed by vancomycin; V+L, vancomycin followed by lysostaphin. Data are shown as mean ± SEM (n=12); ****p<0.0001; ***p<0.001; **p<0.01; *p<0.05.

**Figure 7:**
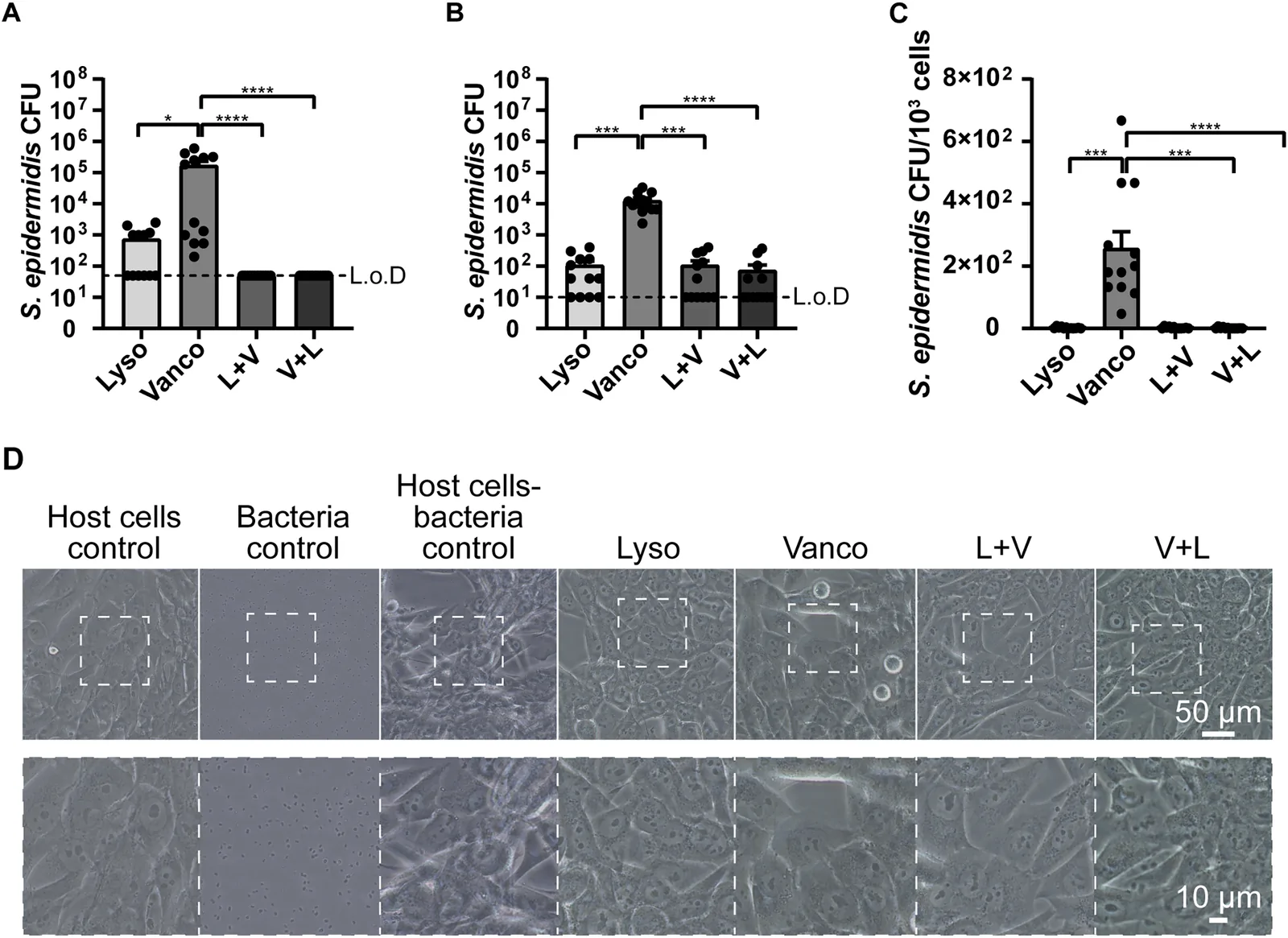
Effect of lysostaphin, vancomycin, and their combination on residual extracellular and cell-associated bacterial recovery of *S. epidermidis* in MG-63 cells. **(A)** Total extracellular CFU. L.o.D.: 50 CFU. **(B)** Total cell-associated CFU from cell lysates. L.o.D.: 10 CFU. **(C)** Cell-associated CFU normalized per host cell (CFU/10^3^ cells). L.o.D.: 0.2 CFU/10^3^ cells. **(D)** Representative phase-contrast microscopy images of live cells at each treatment step; bottom panels correspond to enlargements of the dashed white squares in the top panel. Lyso, lysostaphin; Vanco, vancomycin; L+V, lysostaphin followed by vancomycin; V+L, vancomycin followed by lysostaphin. Data are shown as mean ± SEM (n=12); ****p<0.0001; ***p<0.001; *p<0.05.

#### 3.3.3 Confocal microscopy

To microscopically assess the bacterial internalization following antimicrobial treatment, *S. aureus* and *S. epidermidis* were detected using species-specific antibodies, whereas cellular membranes of both bacteria and host cells were counterstained with a fluorophore-conjugated wheat germ agglutinin (WGA). Representative optical sections and the corresponding whole-volume 3D reconstructions are shown in **Figures 8**, **9**. Confocal imaging qualitatively revealed a greater host-cell-associated burden of *S. aureus* than of *S. epidermidis* in both MG-63 (**Figure 8A**) and hBM-MSCs (**Figure 9A**). In the *S. aureus*-infected cultures, numerous green aggregates were detected within the WGA-defined host-cell volume, frequently clustering in perinuclear regions and, in some cells, extending throughout a large portion of the cytoplasm (**Figures 8A**, **9A**). The 3D reconstructions (**Figures 8B**, **9B**) showed the bacterial signal within the cellular volume and in proximity to host-cell nuclei, supporting an intracellular and predominantly perinuclear localization. By contrast, *S. epidermidis*-infected MG-63 cells (**Figures 8C, D**) and hBM-MSCs (**Figures 9C, D**) displayed substantially fewer green fluorescent puncta and small bacterial aggregates. Only occasional signals were detected within the WGA-defined host-cell volume, while additional sparse puncta were observed in extracellular regions. Compared with the *S. aureus*-infected cultures, the WGA-positive outlines of *S. epidermidis*-infected cells were more readily discernible, and the overall cellular morphology appeared better preserved. Taken together, the confocal images qualitatively support a greater host-cell-associated and intracellular bacterial burden for *S. aureus* than for *S. epidermidis* under the experimental conditions examined. A broader selection of sequences of optical sections and the corresponding whole 3D volume reconstructions are shown in **Supplementary Figures 1**–**8** to allow the qualitative visualization of bacteria inside and outside the cellular volume. Following antimicrobial treatment, bacterial fluorescence was detected mainly in intermediate optical planes and within the WGA-defined host-cell volume, while it decreased or disappeared toward the upper and lower limits of the z-stacks, supporting intracellular localization. This pattern was prominent for *S. aureus* in both MG-63 cells and hBM-MSCs (**Supplementary Figures 1**, **5**), whereas only occasional intracellular *S. epidermidis* signals were observed (**Supplementary Figures 3**, **7**). By contrast, untreated controls showed additional surface-associated and extracellular bacterial signals distributed across a broader range of optical planes (**Supplementary Figures 2**, **4**, **6**, **8**).

**Figure 8:**
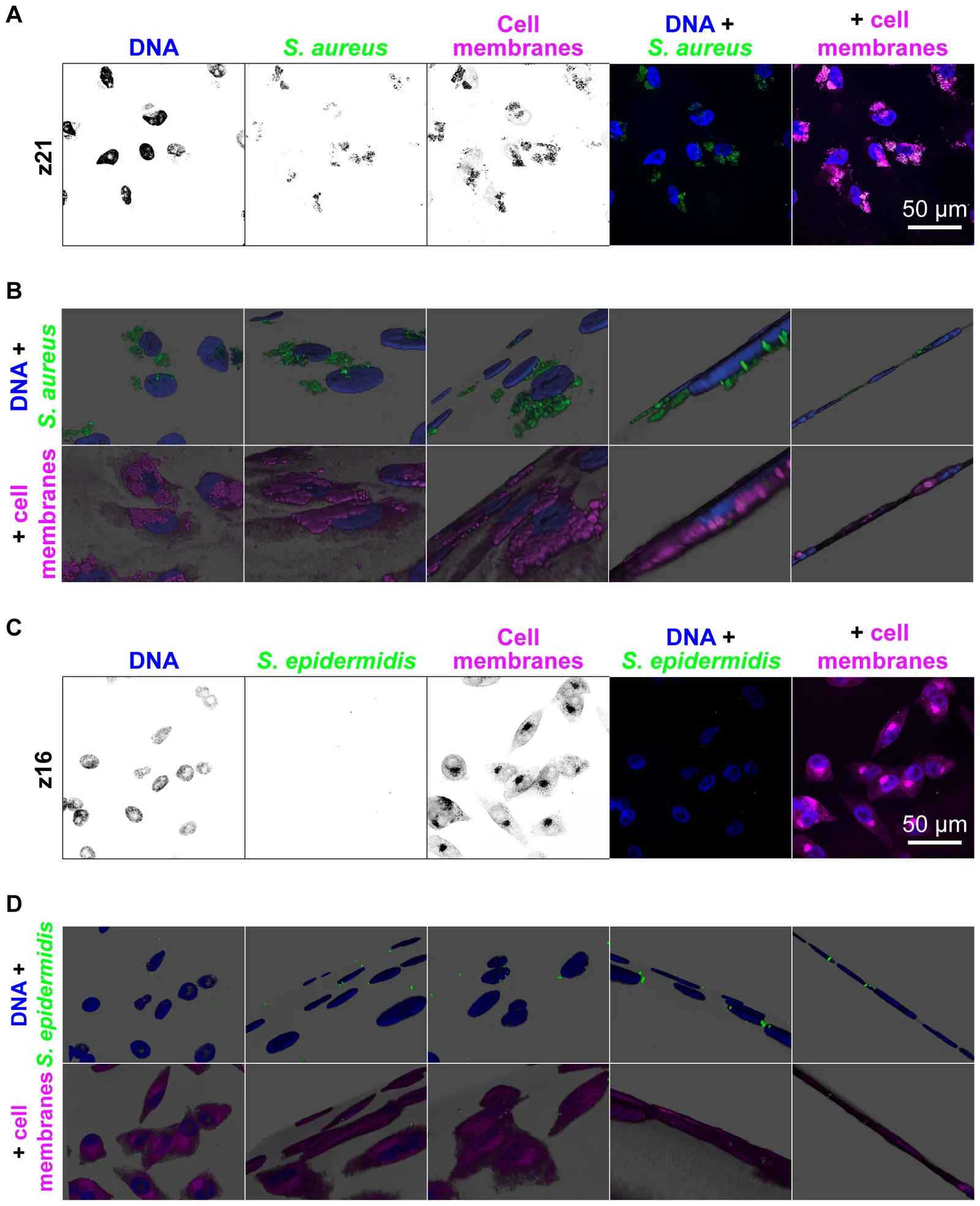
Representative confocal images of MG-63 cells after **(A, B)** *S. aureus* or **(C, D)** *S. epidermidis* infection and subsequent antimicrobial treatment (lysostaphin followed by vancomycin). Blue: DAPI-DNA; green: AF488-antibody-bacteria; magenta: AF647-WGA-cellular membranes. **(A**, **C)** Representative image of a single z-plane corresponding to the central slice of the host cells volume (scale bar 50 µm); single channels are shown in gray scale and as colored overlays of two or three channels. **(B**, **D)** 3D reconstruction of the whole z-stack volume encompassing the host cells, shown from different angles as double- or triple-channel overlay.

**Figure 9:**
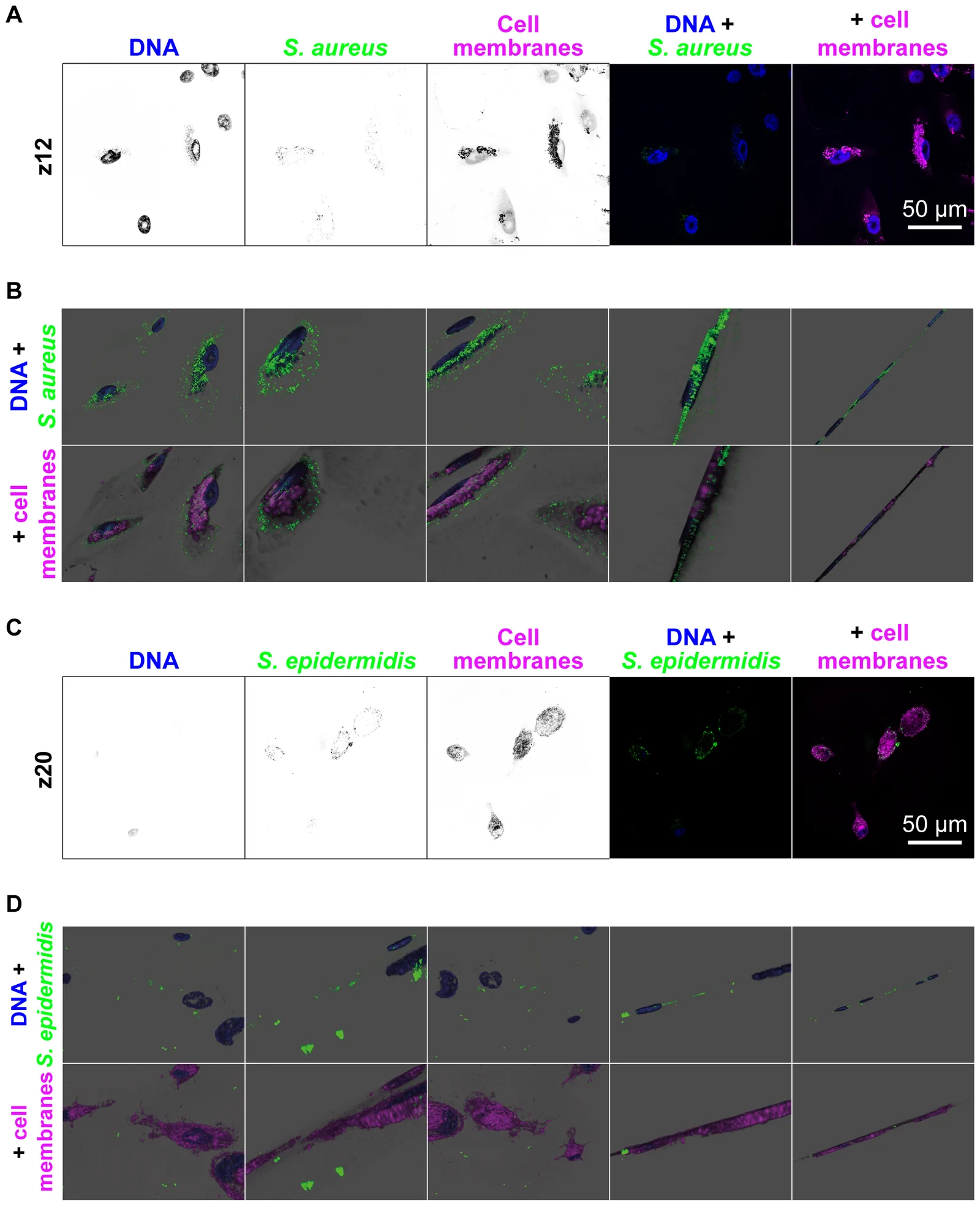
Representative confocal images of hBM-MSCs after **(A, B)** *S. aureus* or **(C, D)** *S. epidermidis* infection and subsequent antimicrobial treatment (lysostaphin followed by vancomycin). Blue: DAPI-DNA; green: AF488-antibody-bacteria; magenta: AF647-WGA-cellular membranes. **(A**, **C)** Representative image of a single z-plane corresponding to the central slice of the host cells volume (scale bar 50 µm); single channels are shown in gray scale and as colored overlays of two or three channels. **(B**, **D)** 3D reconstruction of the whole z-stack volume encompassing the host cells, shown from different angles as double- or triple-channel overlay.

## 4 Discussion

Orthopedic implant-related infections are difficult to eradicate because staphylococci can persist in biofilms and within bone-associated host cells, where they are less susceptible to immune defenses and antimicrobial treatment (Arciola et al., 2018; Josse et al., 2015; Collet et al., 2025). Accurate assessment of this intracellular reservoir requires reliable discrimination between internalized and residual extracellular bacteria. This study developed a protocol for the recovery and quantification of internalized staphylococci in human osteoblast-like MG-63 and primary hBM-MSCs and identified methodological limitations. Our findings show that extracellular-clearance treatments must be validated in the complete bacteria–host-cell system before lysate-derived CFU are interpreted as intracellular. Extracellular bacteria are conventionally eliminated through the gentamicin protection assay (Hamza and Li, 2014) because of its limited short-term penetration into mammalian cells. However, gentamicin intracellular activity can vary and may reduce recovery of internalized staphylococci, leading to underestimation of the viable intracellular population; lysostaphin-based assays have therefore been proposed as an alternative (Kim et al., 2019; Rigaill et al., 2021; Yu et al., 2020). Because *S. epidermidis* was gentamicin-resistant, vancomycin and lysostaphin were evaluated as alternative extracellular-clearance agents. Free vancomycin generally shows limited intracellular efficacy, and cell-penetrating delivery strategies have been developed to improve its activity against intracellular *S. aureus* (Valour et al., 2015; Jiang et al., 2020). Lysostaphin has been used more directly as an extracellular killing agent in staphylococcal invasion and enzyme-protection assays, including osteoblast-related models (Menzies and Kourteva, 1998; Edwards and Massey, 2011; Kim et al., 2019; Valour et al., 2015; Rigaill et al., 2021). The cell-free screening demonstrated that clearance efficacy depended on both bacterial burden and bacterial species. Vancomycin reduced bacterial recovery but did not consistently achieve complete clearance, particularly at the higher simulated MOI. This observation is compatible with previous evidence that vancomycin activity against *S. aureus* can be affected by bacterial inoculum size (LaPlante and Rybak, 2004). However, because MIC values and formal time-kill kinetics were not determined, our results should be interpreted as an inoculum-associated difference in clearance efficacy rather than a change in intrinsic antimicrobial susceptibility. In the presented cell-free assay, lysostaphin was markedly more effective against *S. aureus* than *S. epidermidis*, whereas vancomycin produced a smaller and less species-discriminating reduction. Because these observations were obtained after short-term treatment of a 2-h surface-adherent population, they should be interpreted as strain- and assay-specific rather than extrapolated from studies of mature biofilms. Reducing the simulated MOI to 50 improved extracellular clearance for both strains and produced a more consistent response across treatment conditions. In the cell-free assay, lysostaphin was more effective than vancomycin against *S. aureus*, but less active against *S. epidermidis* and more influenced by bacterial burden. The glycylglycine endopeptidase activity of lysostaphin provides a mechanistic rationale for its extracellular anti-staphylococcal activity, and its ability to disrupt *S. aureus* and *S. epidermidis* biofilms has been demonstrated on artificial surfaces (Wu et al., 2003). However, those mature biofilm conditions differ from the short-term model used here. The species- and inoculum-dependent responses observed in the present study therefore reinforce the need to validate lysostaphin concentration and exposure time within the intended protection assay (Kim et al., 2019; Rigaill et al., 2021). Nevertheless, the species-specific responses indicate that protocols optimized for *S. aureus* may not be equally effective against coagulase-negative staphylococci. Reducing the simulated MOI from 100 to 50 improved clearance consistency and treatment discrimination; therefore, an MOI of 50 was selected for cell-based assays using 25 µg/mL lysostaphin for 30 min and 100 µg/mL vancomycin for 60 min. Validation in infected MG-63 cells and hBM-MSCs showed that vancomycin alone left the highest residual bacterial load in the final treatment medium. Lysate-derived CFU could therefore not be interpreted unambiguously as intracellular, because extracellular or cellular surface-associated bacteria may have contributed to the cell-associated fraction. In contrast, lysostaphin alone and both sequential lysostaphin-containing regimens reduced extracellular recovery to near or below the L.o.D. Based on the results on the extracellular clearance, the CFU counts from cellular lysates are likely composed by both internalized bacteria and: a high fraction of extracellularly adherent bacteria in the case of vancomycin treatment; near-to-complete extracellular clearance in the case of lysostaphin-based treatments. However, since the pure CFU count does not allow to discriminate between extracellularly adherent bacteria and internalized ones, the lysate-derived CFU were always defined as cell-associated CFU. Lysostaphin has similarly been used to remove extracellular staphylococci before assessing internalization in osteoblasts, osteocytes, and other phagocytic cells (Menzies and Kourteva, 1998; Edwards and Massey, 2011; Kim et al., 2019; Yang et al., 2018; Rigaill et al., 2021). The heterogeneity of published intraosteoblastic models further supports model-specific validation of extracellular-clearance conditions (Marro et al., 2021). The three lysostaphin treatments did not, however, yield identical cell-associated recovery in *S. aureus*-infected cultures: the vancomycin followed by lysostaphin treatment (V+L) produced the lowest counts in MG-63 cells, whereas lysostaphin alone produced higher recovery. Because the total post-infection incubation time was not equalized across treatments, these differences cannot be attributed to antimicrobial agent sequence alone. The collection at the end of each respective treatment, rather than uniforming incubation times, is a limitation that prevents further interpretation of the differences observed between the effective lysostaphin-based treatments. Several mechanisms were not evaluated, which may have contributed to these differences, including differential removal of adherent bacteria, host-cell antibiotic uptake, effects on intracellular viability, or bacterial loss during washing. For this reason, in a methodological assay, the preferred agent is the one that removes extracellular bacteria while preserving host-cell integrity. Future studies should consider validation of cellular number and viability after infection and subsequent antimicrobial treatment, not examined here, in order to gain further insights on antimicrobial combinations otherwise giving not significantly different outcomes, such as the lysostaphin-based ones in the present work. Host-cell context may explain why treatment effects were significant in MG-63 but not in hBM-MSC cultures. MG-63 are osteosarcoma-derived osteoblast-like cells, whereas the hBM-MSCs are undifferentiated stromal cells cultured in a different medium; differences in phenotype, adhesion receptors and extracellular matrix, antimicrobial exposure, and susceptibility to infection or detachment may therefore affect extracellular clearance and cell-associated recovery (Hamza and Li, 2014). Pooling three donors reduced variability but prevented assessment of donor-specific effects, a recognized feature of hBM-MSC cultures. Thus, these findings should not be directly extrapolated to primary differentiated osteoblasts, and clearance conditions require validation in each host-cell model. The results also emphasize the need to define MOI experimentally, as published osteoblast models use widely different bacterial burdens and both uptake and host-cell responses depend on MOI and exposure conditions (Hamza and Li, 2014; Bongiorno et al., 2020; Stracquadanio et al., 2021). Here, MOI 50 was not universally optimal but represented a suitable compromise for the tested strains and cell models, maintaining measurable cell-associated bacterial recovery while improving extracellular clearance compared with the higher simulated MOI. After effective extracellular clearance, *S. aureus* remained recoverable from both MG-63 cells and hBM-MSCs, whereas cultivable *S. epidermidis* RP62A was recovered at or near the L.o.D. after lysostaphin-containing treatments. Previous findings are not fully concordant; in fact, Valour and colleagues reported *S. epidermidis* internalization by human osteoblasts (Valour et al., 2013), whereas Campoccia et al. detected no osteoblast invasion by the strains examined (Campoccia et al., 2016). These contrasting observations suggest that bacterial recovery may depend on strain-specific adhesion and biofilm-associated traits, as well as on the host-cell model, infection duration, extracellular-protection procedure, and assay sensitivity (Valour et al., 2013; Campoccia et al., 2016; Stracquadanio et al., 2021; Severn and Horswill, 2023). Accordingly, our results indicate absent or very low recovery of cell-associated *S. epidermidis* under the tested conditions, rather than demonstrating absence of internalization per se. Whenever possible, the intracellular localization of cell-associated bacteria should be independently confirmed by microscopy before lysate-derived CFU are interpreted as intracellular (Hamza and Li, 2014; Bongiorno et al., 2020; Stracquadanio et al., 2021). The confocal and culture-based data indicate that *S. aureus* has a substantially greater capacity than *S. epidermidis* to persist intracellularly in both MG-63 cells and hBM-MSCs after extracellular bacterial killing. The confinement of the residual *S. aureus* signal to the host-cell volume, together with its predominantly perinuclear distribution, supports the interpretation that the CFUs recovered after antimicrobial treatment were consistent with a predominantly intracellular, cell-associated population under the tested conditions rather than surface-associated contaminants. This conclusion is strengthened by the untreated controls, in which bacterial signals showed a broader spatial distribution compatible with the coexistence of extracellular, surface-associated, and intracellular populations. By contrast, the sparse intracellular *S. epidermidis* signal was consistent with the very low CFU values obtained in the internalization assay, suggesting a lower internalization efficiency under the same experimental conditions. This limited internalization is in agreement with previous observations in human osteoblast models (Valour et al., 2013). The reduced definition of stained cellular boundaries in *S. aureus*-infected cultures may reflect a greater perturbation of host-cell architecture associated with the higher bacterial burden. However, confocal morphology alone does not demonstrate loss of membrane integrity. Therefore, this observation should be interpreted as suggestive of infection-associated structural alterations rather than as direct evidence of membrane damage. In contrast, the more clearly preserved cellular outlines observed after *S. epidermidis* infection are consistent with its lower intracellular burden and potentially weaker capability to perturb the membrane stability of the host cells. The results exposed in this study present limitations which the authors acknowledge to provide researchers with further material for troubleshooting issues that might arise when planning and interpreting this kind of experiments. Limitations include the use of a single strain per species and only two bone-derived cell models, the absence of uninfected host-cell controls exposed to antimicrobial treatment, the unequal incubation time and number of washes across the different treatments, the absence of post-treatment and post-infection host-cell counts or viability assays. Finally, only one time point was assessed, without examining later persistence, replication, phenotypic adaptation, or bacterial release, which however would give rise to a whole new separate study and belongs to the outlook of the presented work. In conclusion, the main contribution of this study is a validation rather than a universally applicable extracellular-clearance protocol. Candidate treatments should first be screened at a defined bacterial burden and subsequently validated in the relevant bacteria–host-cell system. Extracellular clearance, host-cell preservation, and recovery of the cell-associated population should be considered separate methodological endpoints. Under the conditions tested, lysostaphin-based treatments were more suitable than vancomycin alone. However, bacterial species and strain, host-cell model, MOI, treatment duration, and washing procedures require re-validation whenever the experimental system is modified.

## Data Availability

The datasets presented in this study can be found in online repositories. The names of the repository/repositories and accession number(s) can be found below: 10.5281/zenodo.21473928.
