# Supplementary material for: A methodological approach to improve extracellular staphylococcal clearance in internalization assays using bone-associated host-cell models of orthopedic infections
Source: Front Cell Infect Microbiol. 2026 Sep 28;16:1945457. doi: 10.3389/fcimb.2026.1945457 (PMC13654847; doi:10.3389/fcimb.2026.1945457)
Supplement: Supplementary file 1 [file Table1.docx]

Supplementary Material

# List of Supplementary Figures

**Supplementary Figure 1.** Representative confocal images of MG-63 cells after *S. aureus* infection and subsequent antimicrobial treatment (lysostaphin followed by vancomycin).

**Supplementary Figure 2.** Representative confocal images of MG-63 cells after *S. aureus* infection without subsequent antimicrobial treatment (bacteria-host-cell control).

**Supplementary Figure 3.** Representative confocal images of MG-63 cells after *S. epidermidis* infection and subsequent antimicrobial treatment (lysostaphin followed by vancomycin).

**Supplementary Figure 4.** Representative confocal images of MG-63 cells after *S. epidermidis* infection without subsequent antimicrobial treatment (bacteria-host-cell control).

**Supplementary Figure 5.** Representative confocal images of hBM-MSC cells after *S. aureus* infection and subsequent antimicrobial treatment (lysostaphin followed by vancomycin).

**Supplementary Figure 6.** Representative confocal images of hBM-MSC cells after *S. aureus* infection without subsequent antimicrobial treatment (bacteria-host-cell control).

**Supplementary Figure 7.** Representative confocal images of hBM-MSC cells after *S. epidermidis* infection and subsequent antimicrobial treatment (lysostaphin followed by vancomycin).

**Supplementary Figure 8.** Representative confocal images of hBM-MSC cells after *S. epidermidis* infection without subsequent antimicrobial treatment (bacteria-host-cell control).

**Supplementary Figure 1.** **Representative confocal images of MG-63 cells after *S. aureus* infection and subsequent antimicrobial treatment (lysostaphin followed by vancomycin).** Blue: DAPI-DNA; green: AF488-antibody-*S. aureus*; magenta: AF647-WGA-cellular membranes. **(A)** Selection of optical planes from a z-stack encompassing the cellular volume (z-step: 0.3 µm; scale bar 50 µm); single channels are shown in grays and as colored overlays of two or three channels. **(B)** 3D reconstruction of the whole z-stack volume, shown by different angles as double- or triple-channel overlay.

**Supplementary Figure 2.** **Representative confocal images of MG-63 cells after *S. aureus* infection without subsequent antimicrobial treatment (bacteria-host-cell control).** Blue: DAPI-DNA; green: AF488-antibody-*S. aureus*; magenta: AF647-WGA-cellular membranes. **(A)** Selection of optical planes from a z-stack encompassing the cellular volume (z-step: 0.3 µm; scale bar 50 µm); single channels are shown in grays and as colored overlays of two or three channels. **(B)** 3D reconstruction of the whole z-stack volume, shown by different angles as double- or triple-channel overlay.

**Supplementary Figure 3.** **Representative confocal images of MG-63 cells after *S. epidermidis* infection and subsequent antimicrobial treatment (lysostaphin followed by vancomycin).** Blue: DAPI-DNA; green: AF488-antibody-*S. epidermidis*; magenta: AF647-WGA-cellular membranes. **(A)** Selection of optical planes from a z-stack encompassing the cellular volume (z-step: 0.3 µm; scale bar 50 µm); single channels are shown in grays and as colored overlays of two or three channels. **(B)** 3D reconstruction of the whole z-stack volume, shown by different angles as double- or triple-channel overlay.

**Supplementary Figure 4.** **Representative confocal images of MG-63 cells after *S. epidermidis* infection without subsequent antimicrobial treatment (bacteria-host-cell control).** Blue: DAPI-DNA; green: AF488-antibody-*S. epidermidis*; magenta: AF647-WGA-cellular membranes. **(A)** Selection of optical planes from a z-stack encompassing the cellular volume (z-step: 0.3 µm; scale bar 50 µm); single channels are shown in grays and as colored overlays of two or three channels. **(B)** 3D reconstruction of the whole z-stack volume, shown by different angles as double- or triple-channel overlay.

**Supplementary Figure 5.** **Representative confocal images of hBM-MSC cells after *S. aureus* infection and subsequent antimicrobial treatment (lysostaphin followed by vancomycin).** Blue: DAPI-DNA; green: AF488-antibody-*S. aureus*; magenta: AF647-WGA-cellular membranes. **(A)** Selection of optical planes from a z-stack encompassing the cellular volume (z-step: 0.3 µm; scale bar 50 µm); single channels are shown in grays and as colored overlays of two or three channels. **(B)** 3D reconstruction of the whole z-stack volume, shown by different angles as double- or triple-channel overlay.

**Supplementary Figure 6.** **Representative confocal images of hBM-MSC cells after *S. aureus* infection without subsequent antimicrobial treatment (bacteria-host-cell control).** Blue: DAPI-DNA; green: AF488-antibody-*S. aureus*; magenta: AF647-WGA-cellular membranes. **(A)** Selection of optical planes from a z-stack encompassing the cellular volume (z-step: 0.3 µm; scale bar 50 µm); single channels are shown in grays and as colored overlays of two or three channels. **(B)** 3D reconstruction of the whole z-stack volume, shown by different angles as double- or triple-channel overlay.

**Supplementary Figure 7.** **Representative confocal images of hBM-MSC cells after *S. epidermidis* infection and subsequent antimicrobial treatment (lysostaphin followed by vancomycin).** Blue: DAPI-DNA; green: AF488-antibody-*S. epidermidis*; magenta: AF647-WGA-cellular membranes. **(A)** Selection of optical planes from a z-stack encompassing the cellular volume (z-step: 0.3 µm; scale bar 50 µm); single channels are shown in grays and as colored overlays of two or three channels. **(B)** 3D reconstruction of the whole z-stack volume, shown by different angles as double- or triple-channel overlay.

**Supplementary Figure 8.** **Representative confocal images of hBM-MSC cells after *S. epidermidis* infection without subsequent antimicrobial treatment (bacteria-host-cell control).** Blue: DAPI-DNA; green: AF488-antibody-*S. epidermidis*; magenta: AF647-WGA-cellular membranes. **(A)** Selection of optical planes from a z-stack encompassing the cellular volume (z-step: 0.3 µm; scale bar 50 µm); single channels are shown in grays and as colored overlays of two or three channels. **(B)** 3D reconstruction of the whole z-stack volume, shown by different angles as double- or triple-channel overlay.
